# Serotype, antimicrobial susceptibility and genotype profiles of *Salmonella* isolated from duck farms and a slaughterhouse in Shandong province, China

**DOI:** 10.1186/s12866-019-1570-z

**Published:** 2019-09-02

**Authors:** Jie Yang, Zijing Ju, Yi Yang, Xiaonan Zhao, Zhiyu Jiang, Shuhong Sun

**Affiliations:** 1 College of Animal Science and Technology, Shandong Provincial Key Laboratory of Animal Biotechnology and Disease Control and Prevention, Shandong Provincial Engineering Technology Research Center of Animal Disease Control and Prevention, Shandong Agricultural University, Daizong Street 61, Tai’an, 271018 China; 2 College of Veterinary Medicine, Yangzhou University, Yangzhou, 225009 China

**Keywords:** *Salmonella*, Antimicrobial resistance, Class I integrons, MLST, PFGE

## Abstract

**Background:**

*Salmonella* has been considered as one of the most important foodborne pathogens that threatened breeding industry and public health. To investigate the prevalence and characterization of *Salmonella* isolated from duck farms and a slaughterhouse in Shandong province, a total of 49 *Salmonella* strains were isolated from 2342 samples from four duck farms and one duck slaughterhouse in Jinan and Tai’an, Shandong province, China.

**Results:**

Among the isolates, *S*. Enteritidis (20/49, 40.8%) and *S*. Anatum (10/49, 20.4%) were the most prevalent, and high resistance rates were detected for erythromycin (49/49, 100.0%) and nalidixic acid (47/49, 95.9%). Class I integrons were detected in 17 isolates (34.7%17/49), which contained gene cassettes *aadA7 + aac3-Id*(15/17) and *aadA5 + dfrA17* (2/17). Eleven different kinds of resistance genes were detected while *bla*_TEM_(36/49, 73.5%) was the most prevalent, followed by *sul2*(14/49, 28.6%). Thirteen virulence genes were tested, and all of the strains carried *invA, hilA* and *sipA*. Multilocus sequence typing (MLST) results showed that seven sequence types (STs) were identified; ST11 was the most prevalent ST (20/49, 40.8%), followed by ST2441 (10/49, 20.4%). There was a strong correlation between STs and serovars. The results of pulsed field gel electrophoresis(PFGE) showed that 39 PFGE patterns were generated from 49 *Salmonella* strains. PFGE patterns were mostly diverse and revealed high similarity between the isolates from the same sampling sites.

**Conclusions:**

The presence of *Salmonella* infections among duck farms revealed that ducks could also be potential reservoirs for *Salmonella*. The high resistance rates against commonly used antimicrobials suggested a need for more reasonable use of antimicrobials, as well as for investigating substitutes for antimicrobials.

## Background

*Salmonella* has been considered to be one of the most important foodborne pathogens associated with public health worldwide, and has been frequently reported in recent years [1, 2], including topics such as its route through the food chain to humans. At present, more than 2600 different serovars have been identified and recorded [3]. In the USA, it is estimated that *Salmonella* causes more than 1 million infection cases annually, resulting in the loss of 365 million dollars (Centers for Disease Control and Prevention, 2014). In China, *Salmonella* infections are also frequently reported, especially among elderly and immunocompromised individuals [4, 5]. In addition, in China, approximately 70 to 80% of outbreaks of foodborne pathogenic diseases are caused by *Salmonella* [6].

In the past decades, the use of antimicrobial agents has been considered the most important way to treat and control *Salmonella* and other pathogens [7]. However, due to widespread utilization of antibiotics, antimicrobial-resistant and even multidrug-resistant *Salmonella* strains have emerged and spread worldwide, and have seriously threatened global public health [8–10].

Many molecular typing techniques are widely used in the field of microbiology and can be used to trace the origins of pathogenic bacteria [11]. Currently, the most widely used molecular typing techniques are MLST and PFGE. The MLST method is convenient and rapid; the resolution is high, and the resulting data is standardly reliable. Through the internet platform, data sharing and comparison between different laboratories is realistic and easier than ever [12]. PFGE is used by laboratories around the world for its high resolution and repeatability, and is widely considered to be the gold standard for molecular typing. However, PFGE does not have a strict, unified international naming standard, and data is not effectively communicated between different laboratories. This study combines two types of typing methods to comprehensively and systematically understand the epidemiological characteristics of *Salmonella*.

According to the FAO (Food and Agriculture Organization) report (2014), China is the largest producer of duck meat, producing 3 million tons annually, and consumption continues to increase every year [13]. Furthermore, Shandong province is China’s largest duck farming province, especially considering that of Tai’an and Jinan. Little information concerning the prevalence and characterization of *Salmonella* from ducks in farms and slaughterhouses in Shandong province is available. Therefore, this study identifies farms and a slaughterhouse as sampling points, analyzing the prevalence and drug resistance of *Salmonella* in these locations; furthermore, these findings may provide beneficial information for the development of the duck industry and public health.

## Methods

### Sample collection

A total of 2342 samples were collected between 2016 and 2017 from Tai’an and Jinan, Shandong province, including samples of duck feces, embryos, livers, intestine and leg meat, in addition to those of feed, drinking water and duck-washing pools and table surfaces (Table 3). All samples were randomly collected, according to the cluster sampling principle, from one duck farm in Jinan (*n* = 450), three duck farms in Tai’an (*n* = 1175) and one slaughterhouse in Tai’an (*n* = 717).In addition, the liver samples were collected from diseased ducks on the three farms, and the samples from the slaughterhouse were collected during the slaughter process. After collection, samples were immediately placed into a sterilized container, then transported to a laboratory, with ice bags, within 6 h for further bacteriological analysis; they were processed immediately upon arrival. Every sampling site was visited only once.

### Isolating and serotyping of *Salmonella*

*Salmonella* strains were isolated from samples using the Chinese National Standard method (GB 4789.4–2010), with some modifications. Briefly, 10.0 mL of buffered peptone water (BPW, Land Bridge Technology, Beijing, China) was added to each sample (1 g) for pre-enrichment. After incubation at 37 °C for 18 h, 1.0 mL pre-enriched culture was inoculated into 10.0 mL selenite cystine broth (SC, Land Bridge Technology) and incubated at 42 °C or 37 °C. After 24 h of incubation, a loop from each broth culture was streaked onto xylose lysinedeoxycholate medium (XLD, Land Bridge Technology) plates and incubated at 37 °C for 24 to 48 h. Next, suspected *Salmonella* colonies were identified by polymerase chain reaction (PCR) assays using primers *invA*. The *invA* gene was a typical violence gene of *Salmonella* that was able to detect and validate *Salmonella* strains with the inclusivity for all subspecies and exclusivity for other genera and species [14]. PCR was performed ina 25.0 μL mixture containing 12.5 μL of 2 × Taq Master Mix (Vazyme Nanjing, China), 9.5 μL ddH_2_O, 1.0 μL of sample DNA, and 1.0 μL of each primer.

All strains were serotyped by slide agglutination using commercial O and H antisera (Tianrun Bio-Pharmaceutical, Ningbo, China) according to the Kauffmann-White scheme.

### Antimicrobial susceptibility testing

The Kirby-Bauer disk diffusion method, as described by the Clinical and Laboratory Standards Institute [15], was used to examine the susceptibility of *Salmonella* to 14 commonly used antibiotics, including ampicillin (AMP; 10 μg), ceftriaxone (CRO; 30 μg), cefotaxime (CTX; 30 μg), erythromycin (EM; 15 μg), chloramphenicol (CHL; 30 μg), florfenicol (FFN; 30 μg), gentamicin (GEN; 10 μg), streptomycin (STR; 10 μg), tetracycline (TET; 30 μg), sulfamethoxazole (SXT; 25 μg), ciprofloxacin (CIP; 5 μg), nalidixic acid (NAL; 30 μg), norfloxacin (NOR; 10 μg), polymyxin B (PB; 300 IU). Meanwhile, *Escherichia coli* strains ATCC 25922 and ATCC 35218 were used as control strains. *Salmonella*isolates resistant to more than three classes of antimicrobials were defined as multidrug-resistance (MDR) isolates [16, 17].

### Detection of antimicrobial resistance genes and virulence genes

Bacterial DNA was extracted using TIANamp Bacterial DNA Kit (TIANGEN, Beijing, China), according to the manufacturer’s instructions. After extraction of DNA, quinolone-resistance genes, including *qnrA*, *qnrB*, *qnrC*, *qnrD*, *qnrS*, *oqxA*and *aac(6′)Ib-cr*; β-lactamase encoding genes, including *bla*_TEM_, *bla*_PSE_, *bla*_CMY-2_, *bla*_SHV_, *bla*_OXA_and *bla*_CTX-M_; aminoglycosides-resistance genes including *aaC1*, *aaC2*,*aaC3*, *aaC4* and *Ant(*2′*)*; tetracycline-resistance genes including *tetA* and *tetB*; sulfonamides-resistance genes, including *sul1*, *sul2* and *sul3*, and chloramphenicol-resistance genes, including *cmlA* and *stcM*, were detected by PCR, using previously described primers (Table 1) and procedures [18–24]. Meanwhile, 13 pairs of primers (Table 2) were used for PCR to detect the virulence genes, including *invA*, *hilA*, *spvC*, *sipA*, *sopE*, *stnP1*, *pefA*, *rck*, *sipC*, *ssaR*, *ssrA*, *sopB* and *sefA* [25]. All of the PCR products were sequenced (Sangon Biotech, Shanghai, China), and the resistance gene subtypes were determined for subsequent analysis.

**Table 1:** Primers used to detect antimicrobial-resistance genes

Resistance Gene Category	Resistance Gene	Primer Sequence	Reference
β-lactamase	*bla*_TEM_	F: 5′- ATAAAATTCTTGAAGACGAAA − 3′	Ahmed et al., 2007 [18]
		R: 5′- GACAGTTACCAATGCTTAATC − 3′	
	*bla*_SHV_	F: 5′- TTATCTCCCTGTTAGCCACC − 3′	Ahmed et al., 2007 [18]
		R: 5′- GATTTGCTGATTTCGCTCGG − 3′	
	*bla*_PSE_	F: 5′- TAGGTGTTTCCGTTCTTG-3′	Puah et al., 2012 [19]
		R: 5′- TCATTTCGCTCTTCCATT-3′	
	*bla*_OXA_	F: 5′- TCAACTTTCAAGATCGCA-3′	Ahmed et al., 2007 [18]
		R: 5′- GTGTGTTTAGAATGGTGA-3′	
	*bla*_CMY-2_	F: 5′- ACGGAACTGATTTCATGATG − 3′	Ahmed et al., 2007 [18]
		R: 5′- GAAAGGAGGCCCAATATCCT −3′	
	*bla*_CTX-M_	F: 5′- CGCTTTGCGATGTGCAG-3′	Ahmed et al., 2007 [18]
		R: 5′- ACCGCGATATCGTTGGT-3′	
Quinolone	*qnrA*	F: 5′- ATTTCTCACGCCAGGATTTG-3′	Ahmed et al., 2007 [18]
		R: 5′- GATCGGCAAAGGTCAGGTCA-3′	
	*qnrB*	F: 5′- GATCGTGAAAGCCAGAAAGG-3′	Ahmed et al., 2007 [18]
		R: 5′- ACGATGCCTGGTAGTTGTCC-3′	
	*qnrC*	F: 5′- GGTTGTACATTTATTGAATC-3′	Ahmed et al., 2007 [18]
		R: 5′- TCCACTTTACGAGGTTCT −3′	
	*qnrD*	F: 5′- AGATCAATTTACGGGGAATA-3′	Ahmed et al., 2007 [18]
		R: 5′- *AACAAGCTGAAGCGCCTG* − 3′	
	*qnrS*	F: 5′- ACGACATTCGTCAACTGCAA-3′	Ahmed et al., 2007 [18]
		R: 5′- TAAATTGGCACCCTGTAGGC-3′	
	*oqxA*	F: 5′- GATCAGTCAGTGGGATAGTTT-3′	Liao et al., 2015 [20]
		R: 5′- TACTCGGCGTTAACTGATTA-3′	
	*aac(6′)-Ib-cr*	F: 5′- TTGCGATGCTCTATGAGTGGCTA − 3′	Ahmed et al., 2007 [18]
		R: 5′- CTCGAATGCCTGGCGTGTTT − 3′	
Aminoglycosides	*aaC1*	F: ACCTACTCCCAACATCAGCC-3′	Navajas-Benito et al., 2016 [21]
		R: ATATAGATCTCACTACGCGC-3′	
	*aaC2*	F:ACTGTGATGGGATACGCGTC-3′	Navajas-Benito et al., 2016 [21]
		R: CTCCGTCAGCGTTTCAGCTA-3′	
	*aaC3*	F: CACAAGAACGTGGTCCGCTA-3′	Navajas-Benito et al., 2016 [21]
		R: AACAGGTAAGCATCCGCATC-3′	
	*aaC4*	F: CTTCAGGATGGCAAGTTGGT-3′	Navajas-Benito et al., 2016 [21]
		R: TCATCTCGTTCTCCGCTCAT-3′	
	*Ant(2′)*	F: ATGTTACGCAGCAGGGCAGTCG-3′	Navajas-Benito et al., 2016 [21]
		R: CGTCAGATCAATATCATCGTGC-3′	
Tetracycline	*tetA*	F: 5′- GCGCCTTTCCTTTGGGTTCT-3′	Navajas-Benito et al., 2016 [21]
		R: 5′- CCACCCGTTCCACGTTGTTA-3′	
	*tetB*	F: 5′- CATTAATAGGCGCATCGCTG-3′	Navajas-Benito et al., 2016 [21]
		R: 5′- TGAAGGTCATCGATAGCAGG-3′	
Sulfonamides	*sul1*	F: 5′- CTTCGATGAGAGCCGGCGGC-3′	Aarestrup et al., 2003 [22]
		R: 5′- GCAAGGCGGAAACCCGCGCC-3′	
	*sul2*	F: 5′- GCGCTCAAGGCAGATGGCATT-3′	Aarestrup et al., 2003 [22]
		R: 5′- GCGTTTGATACCGGCACCCGT-3′	
	*sul3*	F: 5′- AGATGTGATTGATTTGGGAGC-3′	Zhang et al., 2009 [23]
		R: 5′- TAGTTGTTTCTGGATTAGAGCCT-3′	
Chloramphenicol	*cmlA*	F: 5′- TGTCATTTACGGCATACTCG-3′	Guerra et al., 2001 [24]
		R: 5′- ATCAGGCATCCCATTCCCAT-3′	
	*stcM*	F: 5′- CACGTTGAGCCTCTATATGG-3′	Guerra et al., 2001 [24]
		R: 5′- ATGCAGAAGTAGAACGCGAC-3′

**Table 2:** Primers used to detect virulence genes

Virulence Gene	Primer Sequence	Reference
*hilA*	F: 5′- CGTGAAGGGATTATCGCAGT −3′	Fardsanei et al., 2017 [25]
	R: 5′- GTCCGGGAATACATCTGAGC −3′	
*invA*	F: 5′- ACAGTGCTCGTTTACGACCTGAAT − 3′	Fardsanei et al., 2017 [25]
	R: 5′- AGACGACTGGTACTGATCGATAAT − 3′	
*pefA*	F: 5′- TTGCACTGGGTGGTTCTGG − 3′	Fardsanei et al., 2017 [25]
	R: 5′- TGTAACCCACTGCGAAAG − 3′	
*rck*	F: 5′- AACGGACGGAACACAGAGTC − 3′	Fardsanei et al., 2017 [25]
	R: 5′- TGTCCTGACGAAAGTGCATC − 3′	
*sefA*	F: 5′- GCAGCGGTTACTATTGCAGC − 3′	Fardsanei et al., 2017 [25]
	R: 5′- TGTGACAGGGACATTTAGCG − 3′	
*sipA*	F: 5′- CCATTCGACTAACAGCAGCA − 3′	Fardsanei et al., 2017 [25]
	R: 5′- CGGTCGTACCGGCTTTATTA − 3′	
*sipC*	F: 5′- AGACAGCTTCGCAATCCGTT − 3′	Fardsanei et al., 2017 [25]
	R: 5′- ATTCATCCCTTCGCGCATCA − 3′	
*sopB*	F: 5′- CCTCAAGACTCAAGATG − 3′	Fardsanei et al., 2017 [25]
	R: 5′- TACGCAGGAGTAAATCGGTG − 3′	
*sopE*	F: 5′- CGAGTAAAGACCCCGCATAC − 3′	Fardsanei et al., 2017 [25]
	R: 5′- GAGTCGGCATAGCACACTCA − 3′	
*spvC*	F: 5′- ACTCCTTGCACAACCAAATGCGGA − 3′	Fardsanei et al., 2017 [25]
	R: 5′- TGTCTCTGCATTTCGCCACCATCA − 3′	
*ssaR*	F: 5′- GTTCGGATTTGCTTCGG − 3′	Fardsanei et al., 2017 [25]
	R: 5′- TCTCCAGTGACTAACCCTAACCAA − 3′	
*ssrA*	F: 5′- CTTACGATTACGCCATTTACGG − 3′	Fardsanei et al., 2017 [25]
	R: 5′- ATTTGGTGGAGCTGGCGGGACT − 3′	
*stnP1*	F: 5′- TTGTCTCGCTATCACTGGCAACC − 3′	Fardsanei et al., 2017 [25]
	R: 5′- ATTCGTAACCCGCTCTCGTCC − 3′	

### Detection of class I integrons

To investigate the presence of class I integrons, a 25 μL total reaction volume, consisting of 12.5 μL 2 × Taq Master Mix (Vazyme Nanjing, China), 9.5 μL ddH_2_O, 1.0 μL of sample DNA, and 1.0 μL of each pair of primers was prepared for PCR (White et al., 2000). PCR products were purified by a purification Kit and then sequenced (Sangon Biotech, Shanghai, China).

### Multilocus sequence typing (MLST)

Seven housekeeping genes (*aroC*, *dnaN*, *hemD*, *hisD*, *purE*, *sucA*, and *thrA*) were selected for molecular typing of *Salmonella* strains according to the instructions from the University of Warwick (http://mlst.warwick.ac.uk/mlst/). The provided protocols from the MLST homepage were used including PCR conditions and primers (http://mlst.warwick.ac.uk/mlst/dbs/Senterica). PCR products were sequenced by Sangon Biotech (Shanghai, China) and then compared with provided housekeeping genes by Blast+ (Basic Local Alignment Search Tool) [11].

### Pulsed-field gel electrophoresis (PFGE)

PFGE was performed based on the protocol of the Centers for Disease Control and Prevention (CDC) with minor modifications [26]. In brief, isolates were streaked on Luria-Bertani (LB) plates and incubated at 37 °C for 24 h, and *Salmonella* were collected and suspended in a cell suspension buffer (CSB). Subsequently,400.0 μL of cell suspension was transferred to a sterile tube and mixed with 20.0 μL of proteinase K (20.0 mg/mL).400.0 μL of melted 1.0% SeaKem Gold agarose (Lonza, Morristown, NJ, USA) with 1.0% sodium dodecyl sulfate (SDS) was then added to 400.0 μL of the cell suspension. The mixture was poured into the plug molds, cooled down, and then transferred to the lysis solution. After the 2-h lysis, the agarose-embedded DNA was stored in 0.5 × Tris-Borate-EDTA (TBE) at 4 °C. The bacterial cells in the agarose plugs were digested with 50 U of XbaI (TaKaRa, Dalian, China) for 2 h at 37 °C. Digested fragments were resolved in 1.0%SeaKem Gold agarose gel in 0.5× TBE using a ChefMapper electrophoresis system (Bio-Rad, Hercules, CA, USA). After performing electrophoresis at 14 °C for 19 h, the gel was stained with Gel-Red (TIANGEN, Beijing, China), the gel images were obtained by UV trans-illumination (Bio-Rad) and the fingerprinting profiles were analyzed by the BioNumerics Software (Applied Maths, Kortrijk, Belgium). According to the manufacturer’s instructions, the unweighted pair-group method (UPGMA) was performed to generate the dendrogram, with settings of 1.5% position tolerance and 0.5% optimization.

## Results

### Isolation and serotyping of *Salmonella*

A total of 49 *Salmonella* strains were isolated from 2342 samples obtained from four large-scaleduck farms and one slaughterhouse, having an isolation rate of 2.1%. Eleven strains were collected from Farm1 in Tai’an (No.1–11), 10 strains were collected from Farm2 in Tai’an (No.12–21), 8 strains were collected from Farm3 in Tai’an (No.22–29), 18 strains were collected from Farm 4 in Jinan (No. 30–47) and 2 strains were collected from the slaughterhouse in Tai’an (No. 48–49). The prevalence of positive samples was 15.3, 1.9, 1.4, 4.0 and 0.3% in farms 1, 2, 3 and 4 and in the slaughterhouse, respectfully (Table 3).The positive rate for *Salmonella* in duck livers from the diseased duck farms (12.3%, 10/81) was higher than that in duck livers from the slaughterhouse (1.2%, 1/83).In addition, a *Salmonella* strain was also isolated from duck feed.

**Table 3:** Sampling sites and isolation rates and MDR rates

Sampling Sites	Sampling Time	Sample Amount	Positive samples	Total	MDR rate
Tai’an Farm 1	2016	72	Embryos (11/72)	15.3% (11/72)	36.3% (4/11)
Tai’an Farm 2	2017	541	Feces(9/466), Feed(1/41), Drinking water(0/34)	1.9% (10/541)	100% (10/10)
Tai’an Farm 3	2017	562	Feces(4/459), Feed(0/66), Livers(4/37)	1.4% (8/562)	75% (6/8)
Jinan Farm 4	2016	450	Feces(12/406), Livers(6/44)	4.0% (18/450)	83.3% (15/18)
Tai’an Slaughterhouse	2017	717	Leg meat(0/74), Livers(1/83), Water samples from Duck-washing pool(1/29), Cotton swabs from table surface(0/13), Intestine (0/518)	0.3% (2/717)	0% (0/2)

Forty-nine *Salmonella* isolates were divided into 6 serotypes (Table 4), including *S*. Enteritidis (*n* = 20), *S*. Anatum (*n* = 10), *S*. Typhimurium (*n* = 8), *S*.Kentucky (*n* = 5), *S. Indiana* (*n* = 5) and *S*. Montevideo (*n* = 1), while *S*. Enteritidis (40.8%, 20/49) was the predominant one, followed by *S*. Anatum (20.4%, 10/49). The *Salmonella* serotype of Farm 1 was relatively singular, being primarily *S*. Anatum (90.9%). In the four duck farms and the slaughterhouse, *S*. Enteritidis (3/5), *S*. Typhimurium (3/5) and *S. Indiana* (3/5) were widely prevalent serotypes. Among the 6 serotypes in this study, we found that all *S. Indiana* from the three farms were multi-drug resistant, being resistant to at least 12 antibiotics, and also contained the most detected type of resistance genes including *bla*_TEM_, *bla*_OXA_, *bla*_CTX-M_*, sul1*, *sul2*and *aaC4.*

**Table 4:** Serotype distribution of duck *Salmonella* isolates

Serotype	No. of isolates (%)					Total (*n* = 49)
	Farm 1 (*n* = 11)	Farm 2 (*n* = 10)	Farm 3 (*n* = 8)	Farm 4 (*n* = 18)	Slaughterhouse (*n* = 2)	
*S*. Enteritidis	0	0	4 (50.0)	14 (77.8)	2 (100.0)	20 (40.8)
*S.* Anatum	10 (90.9)	0	0	0	0	10 (20.4)
*S*. Typhimurium	0	3 (30.0)	2 (25.0)	3 (16.7)	0	8 (16.3)
*S*. Kentucky	0	5 (50.0)	0	0	0	5 (10.2)
*S. Indiana*	0	2 (20.0)	2 (25.0)	1 (5.6)	0	5 (10.2)
*S*. Montevideo	1 (9.1)	0	0	0	0	1 (2.0)

### Antimicrobial susceptibility testing

**Fig. 1:**
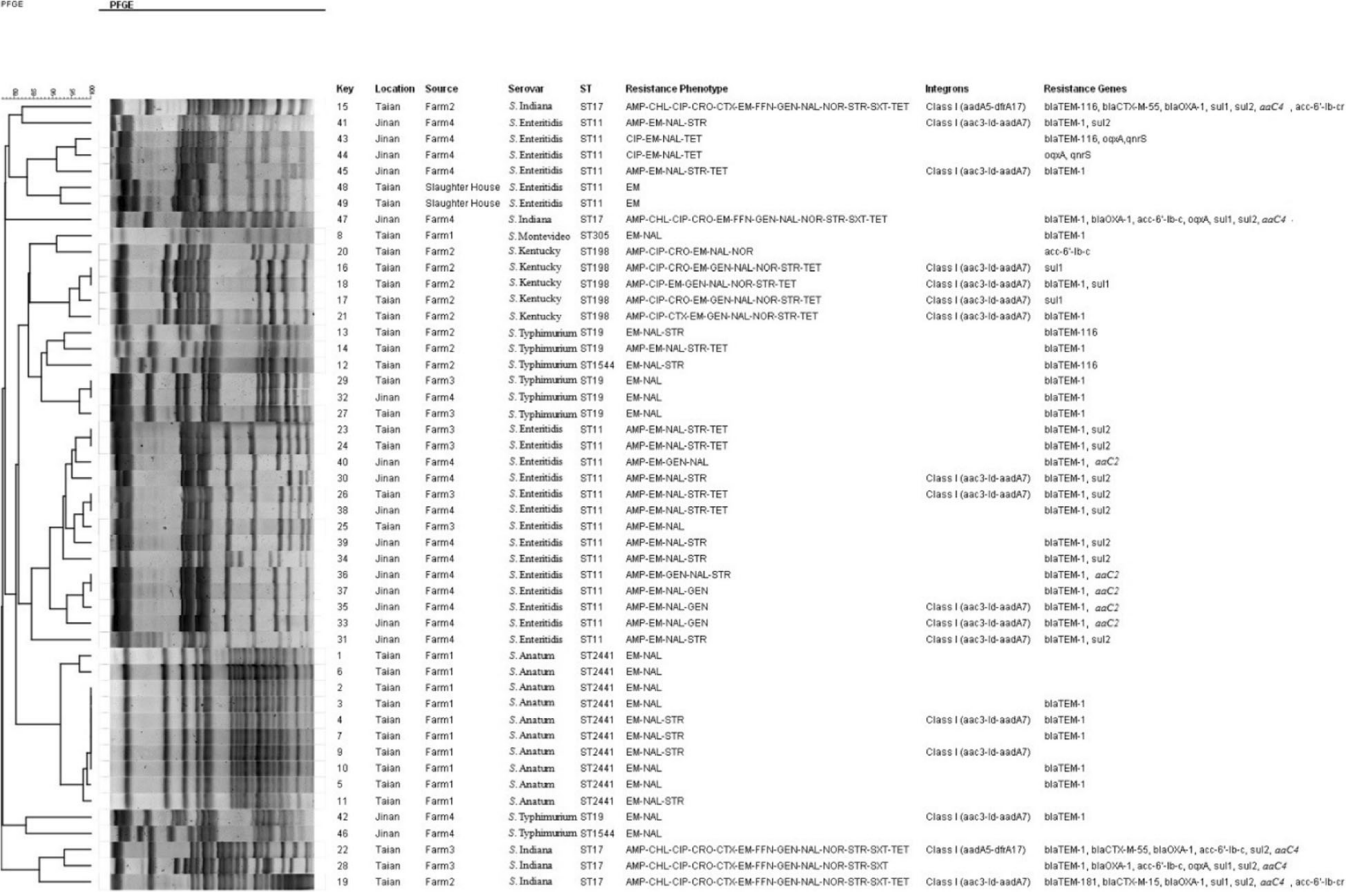
PFGE Dendrogram of 49 *Salmonella* isolates from duck farms and a slaughterhouse in Shandong Province, China

All of the 49 isolated *Salmonella* strains were tested for susceptibility against 14 antimicrobial agents; the results are listed in Table 5. It is noteworthy that 100.0 and 95.9% of those strains were resistant against EM and NAL, respectively, while all of the isolates were sensitive to PB. *Salmonella* isolated from the slaughterhouse were only resistant to EM, compared to *Salmonella* isolated from the farms (Table 6). The most common drug resistance spectrum was EM-NAL (*n* = 12). Among all of the isolates, 35 isolates exhibited multidrug-resistance (MDR), yielding the high rate of 71.4% (Fig. 1).

**Table 5:** Antimicrobial resistance rates for 49 *Salmonella* serovars

Drugs	Enteritidis (*n* = 20)	Anatum (*n* = 10)	Typhimurium (*n* = 8)	Kentucky (*n* = 5)	Indiana (*n* = 5)	Montevideo (*n* = 1)
AMP	80.0	0	12.5	100.0	100.0	0
CRO	0	0	0	60.0	100.0	0
CTX	0	0	0	20.0	80.0	0
EM	100.0	100.0	100.0	100.0	100.0	100.0
GEN	25.0	0	0	80.0	100.0	0
STR	55.0	40.0	37.5	80.0	100.0	0
TET	35.0	0	12.5	80.0	80.0	0
SXT	0	0	0	0	100.0	0
NOR	0	0	0	100.0	100.0	0
CIP	10.0	0	0	100.0	100.0	0
NAL	90.0	100.0	100.0	100.0	100.0	100.0
CHL	0	0	0	0	100.0	0
FFN	0	0	0	0	100.0	0
PB	0	0	0	0	0	0

**Table 6:** Antimicrobial resistance phenotypes of 49 *Salmonella* isolates

Antibacterial agents	Number of resistant isolates (%)					Total (*n* = 49)
	Sample from					
	Farm 1 (*n* = 11)	Farm 2 (*n* = 10)	Farm 3 (*n* = 8)	Farm 4 (*n* = 18)	Slaughterhouse (*n* = 2)	
β-Lactams						
AMP	0	8 (80.0)	6 (75.0)	13(72.2)	0	27 (55.1)
CRO	0	5 (50.0)	2 (25.0)	1 (5.6)	0	8 (16.3)
CTX	0	3 (30.0)	2 (25.0)	0	0	5 (10.2)
Macrolides						
EM	11 (100.0)	10 (100.0)	8 (100.0)	18 (100.0)	2 (100.0)	49 (100.0)
Aminoglycosides						
GEN	0	6 (60.0)	2 (25.0)	6 (33.3)	0	14 (28.6)
STR	4 (36.4)	9 (90.0)	5 (62.5)	9 (50.0)	0	27 (55.1)
Tetracyclines						
TET	0	7 (70.0)	4 (50.0)	5 (27.8)	0	16 (32.7)
Sulfonamides						
SXT	0	2 (20.0)	2 (25.0)	1 (5.6)	0	5 (10.2)
Quinolones						
NOR	0	7 (70.0)	2 (25.0)	1 (5.6)	0	10 (20.5)
CIP	0	7 (70.0)	2 (25.0)	3 (16.7)	0	12 (24.5)
NAL	11 (100.0)	10 (100.0)	8 (100.0)	18 (100.0)	0	47 (95.9)
Amphenicols						
CHL	0	2 (20.0)	2 (25.0)	1 (5.6)	0	5 (10.2)
FFN	0	2 (20.0)	2 (25.0)	1 (5.6)	0	5 (10.2)
Polypeptide						
PB	0	0	0	0	0	0

### Detection of antimicrobial resistance genes and virulence genes

Among the 49 *Salmonella* isolates, 11 kinds of resistance genes were detected (Fig. 1).It is noteworthy that there were 36 strains carrying *bla*_TEM_(73.5%, 36/49), including *bla*_TEM-1_ (*n* = 31), *bla*_TEM-116_ (*n* = 4), and *bla*_TEM-181_ (*n* = 1), and 14 isolates carrying *sul2* (28.6%, 14/49).None of the 49 *Salmonella* strains displayed the tetracycline resistance gene and the chloramphenicol resistance gene. Moreover, there are nine *Salmonella* isolates in which resistance genes were not detected, of which five were collected from Farm1 (Fig. 1). Thirteen virulence genes including *invA*, *hilA*, *spvC*, *sipA*, *sopE*, *stnP1*, *pefA*, *rck*, *sipC*, *ssaR*, *ssrA*, *sopB* and *sefA* were also detected (Table 7).We found that all of the isolates(100.0%, 49/49) carried *invA*, *hilA* and *sipA*, followed by *stnP1* (91.8%, 45/49) and *ssrA* (91.8%, 45/49); only a few isolates carried the *ssaR* (16.3%, 8/49) and *sopE* (16.3%, 8/49) genes.

**Table 7:** Results of detection of virulence genes

Virulence genes	Farm 1 (*n* = 11)	Farm 2 (*n* = 10)	Farm 3 (*n* = 8)	Farm 4 (*n* = 18)	Slaughterhouse (*n* = 2)	Total (*n* = 49)
*invA*	100.0	100.0	100.0	100.0	100.0	100.0
*hilA*	100.0	100.0	100.0	100.0	100.0	100.0
*sipA*	100.0	100.0	100.0	100.0	100.0	100.0
*stnP1*	100.0	100.0	87.5	94.4	0	91.8
*ssrA*	100.0	100.0	87.5	88.9	50.0	91.8
*sipC*	100.0	90.0	50.0	38.9	0	63.3
*spvC*	0	30.0	50.0	77.8	0	42.9
*rck*	0	30.0	62.5	61.1	0	38.8
*sefA*	0	0	50.0	77.8	50.0	38.8
*pefA*	0	30.0	50.0	66.7	0	38.8
*sopB*	45.5	50.0	50.0	22.2	0	36.7
*ssaR*	0	20.0	25.0	22.2	0	16.3
*sopE*	0	0	25.0	33.3	0	16.3

### Detection of class I integrons

Among all of the 49 *Salmonella* strains, 17 strains were found to be carrying class I integrons (Fig. 1), yielding at detection rate of 34.7%. Two kinds of gene cassettes were found in those class Iintegrons, which were *aadA7 + aac3-Id* (15/17, 88.2%) and *aadA5 + dfrA17* (2/17, 11.8%). Furthermore, 94% of *Salmonella* carrying class I integrons were multi-drug resistant in our study.

### MLST

The 49 *Salmonella* isolates were classified into 7 STs (Table 8). The dominant ST isST11 (20/49, 40.8%), followed by ST2441 (10/49, 20.4%), ST19 (6/49, 12.2%) and ST17 (5/49, 10.2%) (Table 8). The STs identified in the present study show the following correlations with *Salmonella* serovars: ST11 with *S.* Enteritidis, ST19 with *S*. Typhimurium, ST17 with *S. Indiana* and ST198 with *S*. Kentucky.

**Table 8:** Prevalence of sequence types (STs) for the *Salmonella* isolates

STs	Serovars	Allelic type							No. of isolates
		aroC	dnaN	hemD	hisD	purE	sucA	thrA	
ST11	Enteritidis	5	2	3	7	6	6	11	19
ST11	Enteritidis	5	2	3	7	6	6	67	1
ST2441	Anatum	10	500	15	31	25	20	33	6
ST2441	Anatum	76	500	15	31	25	20	33	3
ST2441	Anatum	10	500	47	31	25	20	33	1
ST19	Typhimurium	10	7	12	9	5	9	2	5
ST1544	Typhimurium	10	7	12	230	5	9	2	2
ST19	Typhimurium	10	7	12	9	5	6	2	1
ST198	Kentucky	10	14	3	77	64	64	67	3
ST198	Kentucky	8	14	11	77	64	64	67	1
ST198	Kentucky	76	14	3	77	64	64	67	1
ST17	Indiana	8	8	11	11	5	11	15	5
ST305	Montevideo	43	41	16	42	34	13	23	1

### PFGE

As shown in Fig. 1, the PFGE patterns were generally diverse between different sampling sites, and showed similarity values of 80–100% among all of the strains. The 49 *Salmonella* isolates were divided into 39 PFGE patterns, which grouped into ten clusters. Most strains of the same serotype have similar PFGE patterns. However, there are also a few strains of the same serotype that have very different PFGE patterns, for example *S. Indiana* (Key 47 and Key 15). In addition, it is noteworthy that the strains derived from the same farm or slaughterhouse exhibited high similarity, and that some of the isolates from different districts have the same PFGE patterns, as exemplified by two *S*. Typhimurium isolates, Key 29 and Key 32; Key 29 is from Tai’an and Key 32 is from Jinan.

## Discussion

In this study, the total isolation rate of *Salmonella* strains was 2.1% (49/2342), which is significantly less than that (12.2%) in conventional farms in Sichuan province in China [16] and the isolation rates, 2.4 and 7.5%, respectively, in Shandong province, as reported by a study conducted from 2009 to 2012 [10]. However, the isolation rate of *Salmonella* in duck embryos (15.3%) from the diseased duck farms was relatively high. This result was similar to other reports [27], which found the positive rate of *Salmonella* to be 21.1% in dead embryos. The isolation rate of *Salmonella* in duck feces samples (1.8%) collected from the diseased duck farms was lower than that (12.3%) in liver samples, probably due to intermittent detoxification of *Salmonella*; even when the duck is infected with *Salmonella*, the pathogen may not be detectable in the collected fecal samples. In addition, different regions, environmental climates and seasons may also cause differing rates of *Salmonella* isolation.

In our study, the predominant serotype was *S*. Enteritidis (40.8%, 20/49), which is consistent with a study [28], conducted throughout twelve provinces in China, that found the most prevalent serotype among duck farms to be *S*. Enteritidis (36.6%, 15/41). This report differs from those of other countries [1, 8], however, which report that *S*. Typhimurium is the most prevalent serotype in Penang, Malaysia and Korea. The underlying reason may be differences in geography and species isolation among farms. In addition, *S*.Kentucky has been rarely reported in ducks; however, it has been reported in other animals, such as chicken [29, 30], pork [31], beef [32] and rabbit [33]. In this study, we found that *S. Indiana* from the three farms showed a high MDR rate (100%, 5/5) and that our findings concerning the phenomenon of particularly serious drug resistance are similar the corresponding report in China [34]. This may be related to the characteristics of *S. Indiana* itself; most of the *S. Indiana* isolates were resistant to many antibiotics, including streptomycin, tetracycline, chloramphenicol and fluoroquinolones, etc. We have detected Class I integrons and related gene cassettes in 3 out of 5 *S*.indiana isolates, which may be responsible for the MDR (Fig. 1.). Generally, the integrons were located at the conjugative plasmids or at the chromosome within the Salmonella Genome island 1 (SGI1) [35]. However, it has been reported that the *S. Indiana* lacks of SGI1, which was supported by whole genome and PCR analyses [36]. At this stage, the exact mechanisms underlying the antimicrobial resistance of the *S*.indiana may remained to be further studied.

Most *Salmonella* isolates identified in our study showed high resistance to NAL (95.9%) and AMP (55.1%), having resistance rates slightly higher than those seen in the study on ducks in the Sichuan province of China [16], which were reported to be 69.6 and 34.8% to NAL and AMP, respectively. Our corresponding results were also higher than another study conducted in South Korea [37] that reported resistance rates to NAL (73.6%) and AMP (24.0%), suggesting that these drugs may have been widely used in ducks during disease control efforts or prevention. In this study, NAL (95.9%) was obviously higher than CIP (24.5%) in quinolone antibiotic. This may be caused by a significantly greater use of NAL than that of CIP. This result was consistent with a study on ducks in Sichuan province in China [16]. The resistance rate of third-generation cephalosporins to CRO was 16.3%, and that to CTX was 10.2%, which are similar to the reports of another study on ducks in Penang, Malaysia [8]. Our study indirectly proved that third-generation cephalosporins have become the primary drugs for the treatment of *Salmonella*, which is in agreement with another study [38]. In our study, MDR isolate rate of *Salmonella* (71.4%) was similar to another study (73.9%) on ducks in Sichuan province in China [16], but higher than a study (50.5%) on ducks from South Korea [36]. MDR *Salmonella* isolates were frequently observed among the farms in this study; notably, *S. Indiana* isolates were resistant to at least 12 antimicrobials, posing a great risk to public health, should these MDR *Salmonella* isolates be transferable to humans via duck or duck-derived products. Reducing the use of antibiotics in ducks is especially important to limiting the emergence of super-MDR organisms and to maintaining good public health, as well as for other animals.

Concerning the detection of antimicrobial resistance genes, the most prevalent β-lactamase-resistance gene was *bla*_TEM_ (36/49, 73.47%), which is different than reports from Sichuan province that state that the most commonly isolated β-lactamase-resistance gene was *bla*_OXA_ [16].The most common quinolone-resistance genes were *aac(6′)-Ib-cr* (10.2%, 5/49), followed by *oqxA* (8.2%, 4/49); these reports differ from those in Xinjiang province, China that report the most prevalent to be *qnrB* (34.3%) [39]. Those differences may be due to the usage of antimicrobials in different areas of China. Meanwhile, in the present study, there was no tetracycline-resistance gene detected among all strains, while 34.7% (17/49) of strains showed resistance to tetracycline; this could be due to mutations of the resistance strains. Furthermore, there were only a few antimicrobial resistance genes and antibiotics detected in isolates from Farm 1, probably due to fewer parental breeding duck resistance genes and to rational use of antibiotics on this farm. In this study, Class I integrons were detected in 17 *Salmonella* strains out of 49 strains (34.7%), which provided a supplement to the data of *Salmonella* in ducks and was similar to the percentage (31.5%) presented in a study on chicken [40]. Class I integrons were associated with MDR *Salmonella* isolates, a finding which is consistent with other reports [16, 41].

All of the strains carried *invA*, *hilA* and *sipA*, in this study; the high detection rates of these virulence genes have also been observed by other researchers. For instance, it was reported that all 34 S. Enteritidis strains (100.0%) isolated in Iran harbored *invA*, *hilA* and *sipA* genes [25]. Generally, a high detection rate of virulence genes emphasizes the pathogenic potential of these isolates, which may foreshadow severe Salmonellosis and threats to public health [25]. Moreover, *Salmonella* isolated from Farm 1 and the slaughterhouse had multiple virulence genes not detected. This phenomenon is similar to patterns in *Salmonella* resistance and antimicrobial resistance genes detected in our study. We suspect that there may be some connection between them, and propose that further study is necessary.

MLST results revealed that 7 STs were identified in the duck farm and slaughterhouse isolates. Among them, ST11 was the most prevalent ST, which is in agreement with the results of a previous study on ducks [37]. Additionally, the result was in concordance with other reports of ST11 being the predominant ST among *Salmonella* isolates from human and food-producing animals in China [42]; our corroboration of these results further highlight the prevalence of ST11 strains in China. Furthermore, we noticed that no ST40 was detected in our study, which is different from reports of previous studies that found ST40 to be significantly prevalent in slaughterhouse and retail markets in Yangzhou, China. It has also been widely detected in the pig industries of the United States and Europe [6, 16]. This may be due to geographical and environmental differences. In this study, the following correlations between STs and *Salmonella* serovars were founded: ST11 with *S.* Enteritidis, ST2441 with *S*. Anatum, ST19 with *S*. Typhimurium, ST17 with *S. Indiana* and ST198 with *S*. Kentucky. These correlations are consistent with those observed in a previously reported study that found STs and serovars to be tightly connected [6].

With regard to other genotyping methods, whole genome sequencing (WGS) is recently considered as one of the most powerful method to differentiate foodborne microorganisms and determine the genetic relatedness of *Salmonella* isolates, and now WGS is growingly being used by Food Safety Authorities worldwide for outbreak investigation and surveillance [43]. Compared to WGS, PFGE, which used to be known as the golden standard of genotyping, are no longer considered cutting edge but still have been efficient in detecting, investigating and control of foodborne infection outbreaks in the past two decades due to its discriminatory power and reproducibility [44]. In the present study, the phenomenon that the strains derived from the same sampling site exhibited high similarity suggests the possibility of clonal spread; for example, isolates collected from Farm 1 exhibited high genotype similarity. However, the strains isolated from different areas, such as Key 26 (Tai’an Farm 3) and Key 38 (Jinan Farm 4), showed the same PFGE pattern, ST, serovar and resistance profiles; this demonstrates that it is vital to take surveillance and controlling measures to prevent the dissemination of *Salmonella* clones. These results suggest the possibility that *Salmonella* can be transmitted between different farms, a conclusion similar to one proposed in a previous study [45]. Furthermore, we have observed that *Salmonella* isolates with the same serotypes have higher PFGE pattern similarity, that PFGE may be more suitable for *Salmonella* genotyping of the same serotype and that PFGE showed greater power in molecular typing, compared with MLST. For instance, the 49 *Salmonella* isolates were divided into 39 patterns and only 7 STs. However, MLST can be used for typing between different serotypes of *Salmonella*. Therefore, the two types of methods can complement each other, and we can use two types of typing methods for bacterial typing in order to analyze their genetic relationships.

## Conclusions

The prevalence and antimicrobial resistance of *Salmonella* in duck farms are potential risks to public health. The data presented in our study illustrated that several duck farms and a slaughterhouse in the Tai’an and Jinan areas of China are contaminated with *Salmonella* and that antimicrobial resistance and MDR is widespread among the strains. PFGE results revealed that the *Salmonella* strains may have the ability to spread among different areas, as well as the ability to cause clonal spread. It is still necessary and critical to reinforce the surveillance and control of *Salmonella* and to search for a substitution for antimicrobials.

## Data Availability

The data used and analyzed during the present study are accessible from the corresponding author on request.

## Abbreviations

AMP: Ampicillin
BPW: Buffered peptone water
CDCP: Centers for Disease Control and Prevention
CHL: Chloramphenicol
CIP: Ciprofloxacin
CLSI: Clinical and Laboratory Standards Institute
CRO: Ceftriaxone
CSB: Cell suspension buffer
CTX: Cefotaxime
EM: Erythromycin
FAO: Food and Agriculture Organization
FFN: Florfenicol
GEN: Gentamicin
MDR: Multidrug-resistance
MLST: Multilocus sequence typing
NAL: Nalidixic acid
NOR: Norfloxacin
PB: Polymyxin B
PCR: Polymerase chain reaction
PFGE: Pulsed field gel electrophoresis
SC: Selenite cystine broth
SDS: Sodium dodecyl sulfate
STR: Streptomycin
STs: Sequence types
SXT: Sulfamethoxazole
TBE: Tris-Borate-EDTA
TET: Tetracycline
UPGMA: Unweighted pair-group method
XLD: Xylose lysinedeoxycholate medium
